# Mucoscopic Features of Oral Lichen Planus: A Retrospective Comparative Study with Inflammatory Mimickers

**DOI:** 10.3390/diagnostics15091084

**Published:** 2025-04-24

**Authors:** Mihaela Paula Toader, Oana Mihaela Condurache Hritcu, Cristina Colac Botoc, Antonia Elena Hutanu, Catalina Anca Munteanu, Roxana Paraschiva Ciobanu, Stefan Vasile Toader, Alin Gabriel Colac, Victor Vlad Costan, Elena Porumb Andrese, Daciana Elena Branisteanu

**Affiliations:** 1 Discipline of Oral Medicine, Oral Dermatology, Grigore T. Popa University of Medicine and Pharmacy, 16 Universitatii Street, 700115 Iasi, Romania; mihaela.toader@umfiasi.ro (M.P.T.); oana.condurache-hritcu@umfiasi.ro (O.M.C.H.); 2 Dermatology Clinic, University Clinical Railways Hospital, 1 Garabet Ibraileanu Street, 700115 Iasi, Romania; antoniaclivet@yahoo.com (A.E.H.); anca.munteanu2@yahoo.com (C.A.M.); r.p.ciobanu@gmail.com (R.P.C.); 3 Discipline of Physiopathology, Grigore T. Popa University of Medicine and Pharmacy, 16 Universitatii Street, 700115 Iasi, Romania; stefan.toader@umfiasi.ro; 4 Department of Oral and Maxillofacial Surgery, ‘Grigore T. Popa’ University of Medicine and Pharmacy, 700115 Iasi, Romania; colac.alin@yahoo.com (A.G.C.); victorcostan@gmail.com (V.V.C.); 5 Discipline of Dermatology, Grigore T. Popa University of Medicine and Pharmacy, 16 Universitatii Street, 700115 Iasi, Romania; andrese.elena@yahoo.com (E.P.A.); debranisteanu@yahoo.com (D.E.B.)

**Keywords:** oral lichen planus, dermoscopy, mucoscopy, white striae, erosions, vascularity

## Abstract

**Background/Objectives**: Oral lichen planus (OLP) is a chronic inflammatory mucocutaneous disorder with a recognized potential for malignant transformation. While histopathological examination remains the diagnostic gold standard, mucoscopy has emerged as a valuable non-invasive tool for assessing striae patterns, vascular features, and pigmentary alterations. This study aimed to evaluate the mucoscopic characteristics of OLP across different oral mucosal sites and to compare them with other inflammatory oral conditions, assessing their diagnostic relevance. **Methods**: A retrospective comparative study was conducted on 106 patients, including 33 with histopathologically confirmed OLP and 73 with other inflammatory oral conditions (pemphigus vulgaris, chronic cheilitis, hyperplastic oral candidiasis, leukoplakia, squamous cell carcinoma, pachyonychia congenita, morsicatio buccarum). Mucoscopic evaluation focused on the buccal mucosa, vermilion, and lingual mucosa. Features assessed included background color, white striae patterns, vascular morphology, the presence of erosions, and other features like blunting of the lingual papillae and scales on the vermilion. Statistical analysis was carried out using SPSS 29.0. **Results**: Reticular striae were highly specific to OLP, particularly on the buccal mucosa (90.9%, *p* < 0.001). Leukoplakia-like lesions were most prevalent on the lingual mucosa and significantly associated with dotted (*p* = 0.027) and looped vessels (*p* = 0.002). Erosions correlated significantly with both dotted (*p* < 0.001) and linear vessels (*p* = 0.011), especially in lingual and vermilion lesions. In comparison, control group lesions displayed significantly more globular structures (*p* < 0.001), veil-like patterns (*p* < 0.001), and diffuse vascular distributions (*p* = 0.018), particularly in cheilitis and candidiasis cases. **Conclusions**: Mucoscopy reveals distinct site-specific patterns in OLP, supporting its role as a non-invasive diagnostic aid. Comparative analysis highlights its utility in differentiating OLP from other inflammatory oral conditions and in identifying lesions with features suggestive of malignant potential. These findings support the integration of mucoscopy into routine clinical practice and warrant further validation through larger, prospective studies.

## 1. Introduction

Oral lichen planus (OLP) is a rare, chronic inflammatory disorder, with a prevalence ranging between 0.5% and 2.2%, with notable female predilection. It primarily occurs in middle-aged and older adults and is characterized by white, lace-like reticular lesions, most commonly on the buccal mucosa [1,2,3,4].

OLP is classified as an oral potentially malignant disorder, with the highest risk of progression to oral squamous cell carcinoma related to clinically red lesions (erosive or atrophic), location on the borders of the tongue and fewer mucosal sites involved [5]. Recent studies have reported an overall malignant transformation rate of 1.39% for OLP over an average follow-up of 5.8 years, while the erosive subtype showed a significantly higher rate of 5.98% [5,6].

Oral manifestations of lichen planus (LP) may present alongside cutaneous lesions or may be the sole clinical manifestation of the disease. These lesions are thought to result from a T-cell-mediated autoimmune response triggered by various antigens, including dental restorative materials, certain medications (particularly antihypertensive drugs), mechanical trauma, or viral infections such as hepatitis C virus. This immune response induces apoptosis of basal keratinocytes and promotes chronic mucosal inflammation, ultimately contributing to the clinical presentation of the disease [7,8,9,10,11,12].

Clinically, OLP lesions can be divided into six distinct subtypes: reticular, bullous, atrophic, plaque-like, papular, and erosive [13].

The reticular form is the most frequently observed, characterized by interlacing white striae known as Wickham’s striae (WS), which may also be present in cutaneous LP [13]. These asymptomatic lesions can appear anywhere in the oral cavity, but predominantly on the posterior buccal mucosa [14]. In contrast, the erosive form (EOLP), presenting as one or multiple erosions on an erythematous base, often causes pain and difficulty in mastication. EOLP follows a chronic, recurrent course, requiring periodic follow-up. Although malignant transformation in OLP is rare, it remains a clinically significant concern, particularly in the erosive subtype. Transformation is thought to result from chronic mucosal inflammation and sustained immune-mediated epithelial damage, promoting dysplastic changes and, ultimately, progression to oral squamous cell carcinoma (SCC). Clinically, features suggestive of malignant transformation include the emergence of persistent, non-healing ulcers, induration, nodular or exophytic growths, and increased symptom severity. Histopathologically, early malignant changes are marked by epithelial dysplasia, characterized by nuclear pleomorphism, loss of basal cell polarity, abnormal mitotic figures, and architectural disarray, while advanced transformation may exhibit stromal invasion consistent with oral squamous cell carcinoma. These findings highlight the necessity for vigilant follow-up and prompt biopsy of suspicious lesions to ensure early detection and management [15,16,17].

The World Health Organization (WHO) established diagnostic criteria in 1978 to distinguish OLP from clinically similar conditions, which were subsequently revised in 2003 to address the inconsistencies between clinical and histopathological diagnoses [14,18].

Clinical criteria:

1. Bilateral, more or less symmetrical lesions;

2. Presence of a lace-like network of slightly elevated gray-white striae (reticular pattern);

3. Erosive, atrophic, bullous, and plaque-type lesions are considered subtypes only if the reticular lesions are concurrently present elsewhere in the oral mucosa;

4. In cases where the lesions resemble OLP but do not fully meet these criteria, the term “clinically compatible with OLP” should be used.

Histopathological criteria:

1. A well-demarcated, band-like infiltrate of lymphocytes confined to the superficial connective tissue;

2. Evidence of basal cell-layer liquefaction degeneration;

3. Absence of epithelial dysplasia;

4. When histopathologic findings are suggestive but not definite, the term “histopathologically compatible with OLP” should be applied.

A definitive diagnosis of OLP requires the fulfillment of both clinical and histopathological criteria. The term “oral lichenoid lesions” should be used in the following scenarios:

1. Clinically characteristic of OLP but histopathologically only compatible with OLP;

2. Histopathologically characteristic of OLP but clinically only compatible with OLP;

3. Clinically and histopathologically compatible with OLP.

Certain variants of OLP can present diagnostic challenges. The plaque-like form predominantly affects the posterior tongue and buccal mucosa and it may mimic oral leukoplakia. The atrophic form is characterized by diffuse erythematous lesions mixed with reticular features. The bullous form is the least common, presenting as fluid-filled vesicles that can rupture, leading to ulceration and a symptomatology similar to EOLP [19,20]. In ambiguous cases, direct immunofluorescence can be employed to differentiate OLP from autoimmune mucosal disorders, aiding in diagnostic accuracy [21]. Recent advancements in artificial intelligence (AI) technologies have positioned AI as a promising adjunctive tool in the diagnosis of OLP, with targeted training significantly enhancing diagnostic accuracy and efficiency. Nonetheless, variability in performance across platforms and anatomical sites underscores the need for continued optimization and cautious integration into clinical workflows [22,23].

Dermoscopy is a non-invasive, in vivo imaging technique that allows the visualization of intraepithelial and subepithelial structures, otherwise not visible during the clinical examination. It has proven especially valuable in dermatology for the evaluation of pigmented lesions, inflammatory disorders, and early malignancies. This is achieved by using a magnification lens and light to visualize intraepidermal and superficial dermal structures. It employs polarized light or a contact fluid to reduce reflection, achieving magnifications of 10× or higher [24]. Mucoscopy, the application of dermoscopy on mucosal sites, may offer diagnostic clues that support clinical differential diagnosis. As a digital tool, when the dermatoscope is attached to a smartphone for image acquisition and further magnification, mucoscopy contributes to improved diagnostic accuracy, guides biopsy site selection, and facilitates documentation and monitoring over time. Its portability, accessibility, and ability to provide real-time insights make it an increasingly relevant adjunct in the examination and diagnosis of oral mucosal lesions, including oral lichen planus and its mimickers [25]. Mucoscopic features of OLP are not yet standardized and based on case reports and case series. They include a lace-like network of gray-white lines (WS), typically seen around papules or ring-shaped structures. The erosive form presents with an atrophic appearance, characterized by areas of erosion, an erythematous background, and keratotic white striae arranged in a network pattern. Atrophic lesions represent a combination of the reticular and erosive forms, showing striae surrounded by erythematous mucosa. The plaque-like subtype is described mucoscopically as resembling leukoplakia [26,27]. However, because clinical and mucoscopic features may overlap with those of other inflammatory oral conditions such as pemphigus vulgaris, chronic cheilitis, candidiasis, and oral leukoplakia, a comparative analysis is essential. Identifying mucoscopy-specific features of OLP could improve the clinical diagnostic orientation, forgoing the need for ancillary tests to exclude autoimmune conditions that may present with similar clinical features.

While mucoscopy may offer significant diagnostic advantages, histopathological analysis remains the gold standard for confirming the diagnosis of OLP [18].

This study aims to describe the mucoscopic features of OLP and to perform a comparative analysis between OLP and other inflammatory oral conditions on mucoscopy. By evaluating specific patterns such as white lesion morphology, vascular structures and distribution, and other changes across various oral mucosal sites, it seeks to identify distinguishing mucoscopic characteristics of OLP. To our knowledge, this is the first study to include a site-specific comparison between OLP and a control group comprising other inflammatory oral disorders, each diagnosed according to established clinical, histopathological, or specific diagnostic criteria (e.g., mycological examination for candidiasis). This approach enhances the diagnostic utility of mucoscopy in differentiating OLP from its mimickers and contributes to the development of a standardized, non-invasive mucoscopic assessment algorithm to support clinical decision-making and biopsy guidance.

## 2. Materials and Methods

A retrospective, observational, comparative study was conducted on 106 patients. Cases included were collected from admissions to the Department of Dermatology of Clinical Railways Hospital of Iasi, as well as from presentations to the Dermatology Outpatient Department, between January 2021 and December 2024. A total of 106 patients were included. The study group comprised 33 patients with histopathologically confirmed OLP. The control group included 73 patients confirmed with other inflammatory oral conditions—chronic cheilitis [actinic (*n* = 20), exfoliative (*n* = 5), eczematous (*n* = 3)], oral pemphigus vulgaris (PV) (*n* = 15), lip squamous cell carcinoma (*n* = 2), oral leukoplakia (*n* = 2), pachyonychia congenital (*n* = 1), hyperplastic oral candidiasis (*n* = 25), and morsicatio buccarum (*n* = 2). To facilitate the comparative analysis with OLP, mucoscopic features of the following conditions were assessed by anatomical site: cheilitis and squamous cell carcinoma of the lip on the vermilion; PV, morsicatio buccarum, and hyperplastic candidiasis on the buccal mucosa; and hyperplastic candidiasis, oral leukoplakia, pachyonychia congenita, and PV on the lingual mucosa. Several patients with either oral PV or hyperplastic oral candidiasis were considered as controls on multiple mucosal sites. The case number distribution of control group patients according to mucosal site can be found in Table 1.

Inclusion criteria:

- Patients diagnosed clinically and histopathologically with OLP according to the WHO criteria;
- Patients diagnosed with other inflammatory oral conditions according to the individual protocol:
  - Pemphigus vulgaris: diagnosis based on clinical, histopathological, and immunological criteria;
  - Oral leukoplakia: diagnosis based on clinical and histopathological findings;
  - Chronic cheilitis: diagnosis based on clinical and histopathological evaluation;
  - Lip squamous cell carcinoma: diagnosis based on clinical and immunological testing;
  - Hyperplastic candidiasis: diagnosis based on clinical and direct mycological examination and mycological culture;
  - Pachynonychia congenita: diagnosis based on clinical and genetic testing.

Exclusion criteria:

- Patients who did not meet the OLP criteria according to the WHO criteria;
- Patients diagnosed with oral lichenoid reactions;
- Patients with only clinical suspicion of other inflammatory oral conditions, lacking immunological or histopathological confirmation.

Each patient underwent a comprehensive intraoral examination, focusing on three primary anatomical sites: buccal mucosa, vermilion border of the lips, and lingual mucosa.

Mucoscopic evaluations were performed using a handheld polarized dermatoscope (Dermlite DL4 and Dermlite DL5, 3Gen, San Juan Capistrano, CA, USA) with 10× magnification, attached to a smartphone via a magnetic adapter, with additional 5× magnification for image acquisition and storage. A non-contact technique using polarized light was employed for all examinations in order to avoid blunting of the lingual papillae or altering the appearance of vascular structures. The following features were recorded for each lesion:

1. Background color—pink, erythematous, violaceous;

2. White lesions—radial striae, linear striae, reticular striae, annular striae, leaf-venation, globular, dotted, veil, rosette, leukoplakia-like (the term is used to describe the mucoscopic appearance of the lesion);

3. Vascular patterns—linear, dotted, looped, sea anemone-like;

4. Distribution of vascular structures—radial at the periphery or diffuse regular;

5. Presence/absence of erosions;

6. Other features—scaling, blunting of lingual papillae.

Mucoscopic images were taken for each mucosal site and analyzed by two dermoscopists. The results are presented comparatively, emphasizing the differences and similarities in mucoscopic findings between OLP and the other evaluated inflammatory oral conditions.

### Statistical Analysis

The statistical analysis was carried out in SPSS 29.0. Data were recorded and analyzed using descriptive and inferential statistical methods. Categorical variables were expressed as frequencies and percentages. To assess potential associations between white lesions and vascular patterns, Pearson’s Chi-square test was performed. A *p*-value < 0.05 was considered statistically significant and a *p*-value < 0.01 was considered statistically highly significant.

## 3. Results

### 3.1. Demographic Characteristics of the Study Group

The study group included a total of 33 patients diagnosed with OLP, with a female predominance (72.2%, *n* = 24/33) compared to males (27.3%, *n* = 9/33). The mean age of the study population was 53.03 years (SD = 15.52), with a median age of 53 years. Female patients had a higher mean age (54.71 ± 14.81 years) compared to males (48.56 ± 17.37 years) (Table 2).

The clinical presentation of OLP in the study cohort predominantly included the reticular form (54.5%, *n* = 18/33), with the erosive form observed in 45.5% (*n* = 15/33). Concomitant cutaneous involvement was noted in 42.4% (*n* = 14/33) of cases, whereas 57.5% (*n* = 19) showed lesions limited to the oral mucosa (Table 3).

### 3.2. Descriptive Analysis of Mucoscopic Features of OLP

To better illustrate the mucoscopic profile within the OLP cohort, we analyzed the overall frequency of each feature across the 33 patients, acknowledging that multiple features could be present in the same individual. The most common background color observed was pink (96.9%, *n* = 32/33), followed by erythematous (51.5%, *n* = 17/33) and violaceous (33.3%, *n* = 11/33) tones. Among white lesions, reticular striae were most prevalent (93.9%, *n* = 31/33), followed by linear striae (84.8%, *n* = 28/33), leukoplakia-like lesions (60.6%, *n* = 20/33), and veil-like patterns (57.5%, *n* = 19/33). Less frequently observed patterns included leaf venation (21.2%, *n* = 7/33), annular striae (15.1%, *n* = 5/33), globular structures (18.1%, *n* = 6/33), dotted white areas (15.1%, *n* = 5/33), and rosettes (3.0%, *n* = 1/33).

In terms of vascular features, dotted vessels were the most frequent (69.6%, *n* = 23/33), followed by linear (57.5%, *n* = 19/33) and looped vessels (24.2%, *n* = 8/33), with sea anemone-like patterns observed in one case (3.0%, *n* = 1/33). The distribution of vessels was most often radial at the periphery (63.6%, *n* = 21/33), while a diffuse regular pattern was observed in 27.3% (*n* = 9/33) of cases. Additional findings included erosions in 48.4% of patients (*n* = 16/33), scales in 42.4% (*n* = 14/33), and blunting of lingual papillae in 60.6% of cases (*n* = 20/33). A schematic distribution of mucoscopic features of the OLP study cohort is summarized in Table 4 and Table 5.

The predominant background color observed in mucoscopic examination varied across anatomical regions. A pink background was the most common, observed in 69.7% of lesions on the vermilion (*n* = 23/33), 63.6% of lingual lesions (*n* = 21/33), and 90.9% (*n* = 30/33) of buccal mucosa lesions. An erythematous background was present in 30.3% (*n* = 10/33) of vermilion lesions, 33.3% (*n* = 11/33) of lingual lesions, and 24.2% (*n* = 8/33) of buccal lesions. A violaceous background was detected in 27.3% (*n* = 9/33) of vermilion lesions but was rarely observed in lingual mucosa (9.1%) (*n* = 3/33) and absent in buccal mucosa (Table 5).

White structures were present in various patterns. Reticular striae were most frequently found on the buccal mucosa (90.9%, *n* = 30/33), while they were less prevalent on the vermilion (39.4%, *n* = 13/33) and the lingual mucosa (18.2%, *n* = 6/33). Linear striae were predominant on the vermilion (81.8%, *n* = 27/33) but were less common on the lingual (12.1%, *n* = 4/33) and buccal mucosa (21.2%, *n* = 7/33). Radial striae were most frequently observed on the buccal mucosa (21.2%, *n* = 7/33), followed by the vermilion (15.2%, *n* = 5/33) and lingual mucosa (9.1%, *n* = 3/33). Annular striae were rare, occurring in 9.1% (*n* = 3/33) of vermilion lesions, 6.1% (*n* = 2/33) of lingual lesions, and 3.0% (*n* = 1/33) of buccal lesions. Globular patterns were identified in 6.1% (*n* = 2/33) of vermilion lesions, 12.1% (*n* = 4/33) of lingual lesions, and 3.0% (*n* = 1/33) of buccal lesions. Leukoplakia-like patterns were more frequently detected on the lingual mucosa (48.5%, *n* = 16/33) compared to the vermilion (18.2%, *n* = 6/33) and buccal mucosa (15.2%, *n* = 5/33) (Table 5).

Dotted blood vessels were the most frequently encountered vascular feature, observed in 57.6% (*n* = 19/33) of vermilion lesions, 36.4% (*n* = 12/33) of lingual lesions, and 15.2% (*n* = 5/33) of buccal lesions. Linear vessels were more frequently seen on the vermilion (39.4%, *n* = 13/33) than on the buccal mucosa (15.2%, *n* = 5/33). Looped vessels were identified in 15.2% (*n* = 5/33) of vermilion lesions, 12.1% (*n* = 4/33) of lingual lesions, and 6.1% (*n* = 2/33) of buccal lesions. Radial peripheral vascular distribution was noted in 48.5% (*n* = 16/33) of lingual and vermilion mucosa lesions, whereas it was less frequent on the buccal mucosa (21.2%, *n* = 7/33). A diffuse regular vascular pattern was present in 30.3% (*n* = 10/33) of vermilion lesions, 6.1% (*n* = 2/33) of lingual lesions, and 6.1% (*n* = 2/33) of buccal mucosa lesions. The rare sea anemone-like vascular pattern was noted in 3.0% (*n* = 1/33) of lingual and buccal mucosa lesions (Table 4 and Table 5).

Erosions were observed in 39.4% (*n* = 13/33) of vermilion lesions, 42.4% (*n* = 14/33) of lingual lesions, and 36.4% (*n* = 12/33) of buccal mucosa lesions. Scaling was noted exclusively on the vermilion (48.5%, *n* = 16/33). Blunting of lingual papillae was observed in 60.6% (*n* = 20/33) of lingual lesions (Table 4 and Table 5).

Statistical analysis revealed significant associations between certain white or erosive lesions and vascular features (Appendix A):

1. Leukoplakia-like lesions were significantly associated with dotted blood vessels (*p* = 0.027), suggesting increased vascularization in hyperkeratotic lesions.

2. A significant association was found between the presence of erosions and dotted blood vessels (*p* < 0.001), as well as between erosions and linear vessels (*p* = 0.011), indicating a stronger angiogenic component in erosive OLP.

3. A trend toward significance was noted between annular striae and dotted blood vessels (*p* = 0.053) and between radial striae and linear vessels (*p* = 0.057).

4. Leukoplakia-like lesions were also significantly associated with looped vessels on the lingual mucosa (*p* = 0.002) and dotted vessels in the buccal mucosa (*p* = 0.044), reinforcing their potential role in advanced disease stages.

### 3.3. Comparative Analysis of Mucoscopic Features According to Mucosal Sites

#### 3.3.1. Vermilion

On the vermilion, an erythematous background was significantly more common in the control group (80.0%, *n* = 24/30) compared to the OLP group (30.3%, *n* = 10/33) (*p* < 0.001), whereas the violaceous background showed no significant difference (27.3%, *n* = 9/33 vs. 30.0%, *n* = 9/30, *p* = 0.811).

**Figure 1:**
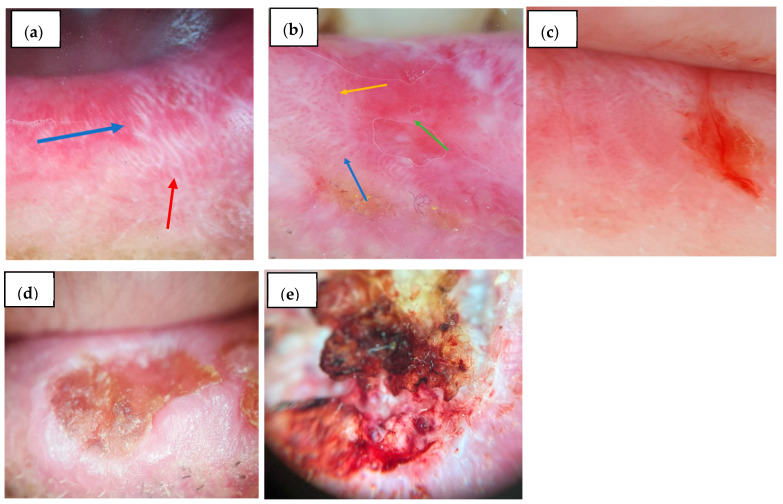
Mucoscopic features on the vermilion: (**a**) OLP: Blue arrow—white linear striae; Red arrow—reticular white striae; (**b**) OLP: Blue arrow—linear striae; Orange arrow—dotted vessels; Green arrow—erosion; (**c**) Eczematous cheilitis—dotted vessels, white veil, scales, and an erosion on a pink background; (**d**) Actinic chronic cheilitis—leukoplakia-like lesion and erosions; (**e**) SCC—erosion surrounded by polymorphous vessels; leukoplakia-like lesion, crusts.

Regarding white lesions, radial striae were observed in 15.2% (*n* = 5/33) of OLP cases and 13.3% (*n* = 4/30) of controls (*p* = 1.000), while linear striae (Figure 1a,b) were present in 81.8% (*n* = 27/33) of OLP patients and 66.7% (*n* = 20/30) of controls (*p* = 0.168). Reticular striae (Figure 1a) were detected exclusively in OLP cases (39.4%, *n* = 13/33, *p* < 0.001). Leaf venation and annular striae were rare and found only in OLP (3.0%, *n* = 1/33 and 9.1%, *n* = 3/33, respectively), but without statistical significance. Globular structures were significantly more frequent in the controls (30.0%, *n* = 9/30) than in OLP (6.1%, *n* = 2/33, *p* = 0.012), and dotted structures were more common in controls (20.0%, *n* = 6/30) than OLP (3.0%, *n* = 1/33), although not statistically significant (*p* = 0.052). The veil pattern was markedly more frequent in the controls (66.7%, *n* = 20/30) compared to OLP (15.2%, *n* = 5/33, *p* < 0.001), and leukoplakia-like lesions were also more prevalent among the controls (50.0%, *n* = 15/30) than OLP patients (18.2%, *n* = 6/33, *p* = 0.007). Rosettes were rare and identified only in one OLP case (3.0%, *n* = 1/33).

Linear and dotted blood vessels (Figure 1b) were comparably distributed in both groups (39.4%, *n* = 13/33 vs. 40.0%, *n* = 12/30, *p* = 0.961 and 57.6%, *n* = 19/33 vs. 76.7%, *n* = 23/30, *p* = 0.108, respectively). Looped vessels showed no significant difference (15.2%, *n* = 5/33 vs. 13.3% *n* = 4/30, *p* = 1.000), and sea anemone–like vessels were observed in only one control subject (3.3%, *n* = 1/30). Radial distribution of vessels at the periphery was seen in 48.5% (*n* = 16/33) of OLP and 30.0% (*n* = 9/30) of controls (*p* = 0.134), while diffuse regular vascular distribution was significantly more frequent in the controls (60.0%, *n* = 18/30) than in OLP (30.3%, *n* = 10/33, *p* = 0.018). Erosions were observed in 39.4% (*n* = 13/33) of OLP (Figure 1b) and 53.3% (*n* = 16/30) of controls (*p* = 0.268) (Figure 1c–e). Scales were significantly more common in controls (100%, *n* = 3/300) than in OLP (48.5%, *n* = 16/33, *p* < 0.001). Comparative mucoscopic features between OLP and controls on the vermilion are presented in Table 6.

#### 3.3.2. Lingual Mucosa

**Figure 2:**
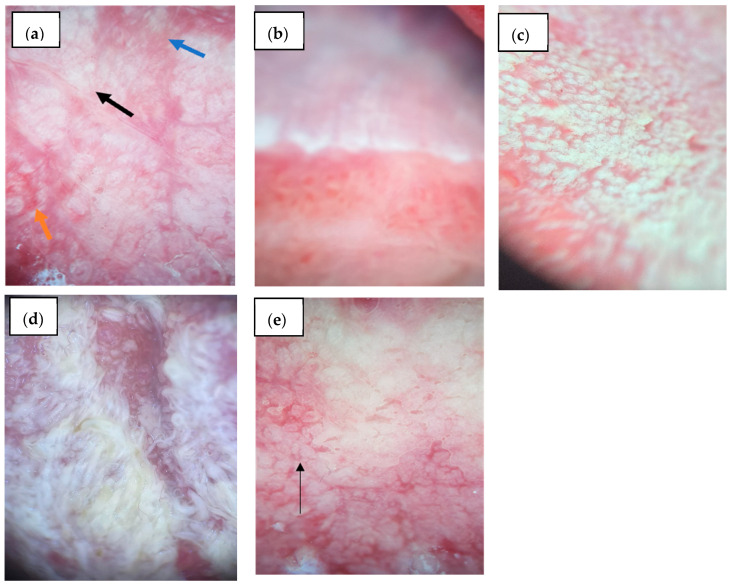
Mucoscopic aspects on the lingual mucosa: (**a**) OLP: Blue arrow—linear striae; OLP: Black arrow—blunted lingual papillae; Orange arrow—dotted vessels; (**b**) Leukoplakia—white structureless homogenous area; (**c**) Oral hyperplastic candidiasis—white hypertrophic filiform papillae; (**d**) Pachyonychia congenita—elongated white-yellow filiform papillae; (**e**) OLP: Black arrow- blunted lingual papillae.

Regarding the lingual lesions, an erythematous background was more frequent in the controls (70.0%, *n* = 21/30) than OLP patients (33.3%, *n* = 11/33, *p* = 0.004), while a violaceous background was present in 9.1% (*n* = 3/33) of OLP cases and absent in controls (*p* = 0.240). Radial striae (9.1%, *n* = 3/33 vs. 3.3%, *n* = 1/30, *p* = 0.614), linear striae (12.1%, *n* = 4/33, vs. 6.7%, *n* = 2/30, *p* = 0.674) (Figure 2a), and reticular striae (18.2%, *n* = 6/33, vs. 3.3%, *n* = 1/30, *p* = 0.107) were slightly more common in OLP but without statistical significance. Annular striae were found in 6.1% (*n* = 2/33) of OLP cases and none of the controls (*p* = 0.493), while leaf venation was absent in both groups. Globular structures were significantly more prevalent in controls (93.3%, *n* = 28/30) compared to OLP (12.1%, *n* = 4/33, *p* < 0.001), while dotted structures were rare in both groups (3.0%, *n* = 1/33 vs. 3.3%, *n* = 1/30, *p* = 1.000). Veil pattern was more common in OLP (42.4%, *n* = 14/33) than controls (20.0%, *n* = 6/30, *p* = 0.056). Leukoplakia-like lesions were found in 48.5% (*n* = 16/33) of OLP (Figure 2a) and 30.0% (*n* = 9/30) of controls (*p* = 0.134) (Figure 2b–d). Rosettes were not observed.

**Figure 3:**
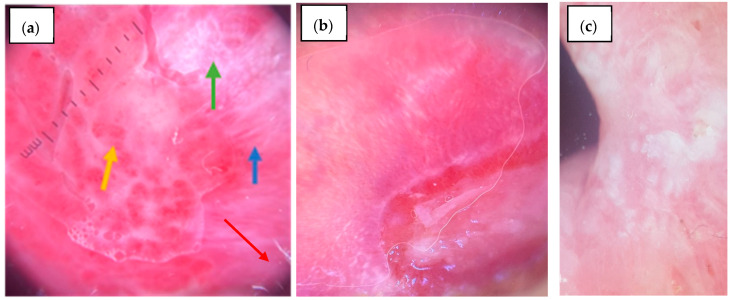
Mucoscopic features on the buccal mucosa: (**a**) OLP: Blue arrow—linear striae; Green arrow—reticular striae; Red arrow—white veil; Yellow arrow—dotted vessels; (**b**) PV—well demarcated erosion, scales, white parallel striae at the periphery of the erosion; (**c**) Morsicatio buccarum—white elevated structureless area, white corrugated bands.

Linear and dotted vessels were significantly more common in OLP (36.4% each, *n* = 12/33) (Figure 3) compared to controls, where they were absent or rare (0% and 3.3%, *n* = 0/30 and *n* = 1/30, respectively; *p* < 0.001 and *p* = 0.001). Looped vessels (12.1%, *n* = 4/33, 0% controls, *p* = 0.115) and sea anemone-like vessels (3.0%, *n* = 1/33, 0% controls, *p* = 1.000) were infrequent. Radial vascular distribution was present exclusively in OLP patients (48.5%, *n* = 16/33, *p* < 0.001), while diffuse regular distribution was seen in 6.1% (*n* = 2/33) of OLP and 3.3% (*n* = 1/30) of controls (*p* = 1.000). Erosions were significantly more common in OLP (42.4%, *n* = 14/33) than controls (16.7%, *n* = 5/30, *p* = 0.026). Scales were present in only 6.7% (*n* = 2/30) of controls and absent in OLP (*p* = 0.223). Blunting of lingual papillae was notably more frequent in OLP (60.6%, *n* = 20/33) (Figure 2e) versus controls (10.0%, *n* = 3/30, *p* < 0.001). Comparative mucoscopic features between OLP and controls on the lingual mucosa are presented in Table 7.

#### 3.3.3. Buccal Mucosa

As for the buccal mucosa lesions, an erythematous background was significantly more common in controls (85.0%, *n* = 17/30) compared to OLP patients (24.2%, *n* = 8/30, *p* < 0.001), while a violaceous background was not observed in either group. Radial striae were present in 21.2% (*n* = 7/33) of OLP and 25.0% (*n* = 5/30) of controls (*p* = 0.748), and linear striae were significantly more frequent in the controls (90.0%, *n* = 18/30) than OLP (21.2%, *n* = 7/33, *p* < 0.001) (Figure 3). Reticular striae were highly specific for OLP, being present in 90.9% (*n* = 30/33) of cases versus only 5.0% (*n* = 1/30) of controls (*p* < 0.001). Leaf venation was found in 18.2% (*n* = 6/33) of OLP and absent in controls (*p* = 0.072). Annular striae were observed in 3.0% (*n* = 1/33) of OLP and none in controls (*p* = 1.000). Globular structures were significantly more common in controls (75.0%, *n* = 15/30) than OLP (3.0%, *n* = 1/33, *p* < 0.001). Dotted structures were also more frequent in the controls (45.0%, *n* = 9/30) than OLP (9.1%, *n* = 3/30, *p* = 0.005), as was the veil pattern (55.0%, *n* = 11/30 vs. 21.2%, *n* = 7/33, *p* = 0.012) (Figure 3a,b). Leukoplakia-like lesions were observed in 30.0% (*n* = 6/30) of controls and 15.2% (*n* = 5/33) of OLP (*p* = 0.296) (Figure 3c). Rosettes were absent in both groups.

Among blood vessel morphologies, linear vessels were observed in 15.2% (*n* = 5/33) of OLP and 5.0% (*n* = 1/30) of controls (*p* = 0.390), and dotted vessels were significantly more frequent in the controls (65.0%, *n* = 13/30) than OLP (15.2%, *n* = 5/33, *p* < 0.001) (Figure 3a). Looped vessels were found in 6.1% (*n* = 2/33) of OLP and 5.0% (*n* = 1/30) of controls (*p* = 1.000). Sea anemone-like vessels were seen in one OLP patient (3.0%, *n* = 1/33). Radial peripheral vascular distribution was observed only in OLP (21.2%, *n* = 7/33, *p* = 0.037), while diffuse regular distribution was significantly more common in controls (65.0%, *n* = 13/30) than OLP (6.1%, *n* = 2/33, *p* < 0.001). Erosions were significantly more frequent in the controls (75.0%, *n* = 15/30) than OLP (36.4%, *n* = 12/33, *p* = 0.006), mainly because our control group consisted, for the most part, of pemphigus vulgaris with active lesions. Comparative mucoscopic features between OLP and controls on the buccal mucosa are presented in Table 8.

## 4. Discussion

Lip localization of LP lesions is quite rare, and the available literature does not provide sufficient mucoscopic patterns to draw a definitive conclusion in order to achieve a final diagnosis solely based on mucoscopy. Clinically, OLP on the lip can present as an erosive or papular form. The background color is reported to appear violaceous or erythematous, with WS in three different patterns: circular, radial, and linear, although isolated cases with different configurations have been mentioned (e.g., leaf venation-like). Additionally, diffuse scaling, pigmentation (in a streak or globule pattern), and various vascular arrangements (most frequently described as linear, hairpin, or dotted) have been identified in the few cases documented in the literature. There are also reports of rosettes identified at the lip margin, alongside erosions and bleeding spots. However, it is considered that the hallmarks of lip LP are represented by a mixture of WS, pigmentation, telangiectasia, and scales [28,29,30]. Our study aligns with previous findings, emphasizing linear striae as the most prevalent white lesion pattern on the vermilion and dotted vessels as the most frequently observed vascular feature. A radial peripheral vascular distribution was observed in 48.5% (*n* = 16/33) of OLP cases on the vermilion, which may serve as a useful differentiating pattern from other inflammatory lip conditions. When compared to the control group, several key differences emerged. In the control cases, globular structures, veil-like appearances, and a diffuse regular vascular distribution were significantly more prevalent. Moreover, scales were universally present in the control group, compared to 48.5% (*n* = 16/33) in OLP. These differences suggest that the combination of linear striae, dotted vessels, and radial vessel distribution may provide diagnostic specificity for OLP, especially in distinguishing it from chronic cheilitis. Erosions were observed in 39.4% (*n* = 13/33) of the vermilion lesions in OLP, and the background was most commonly pink, followed by erythematous and violaceous, which may reflect inflammation and vascular involvement.

The buccal mucosa is a common site for lesions of OLP and has drawn significant attention for mucoscopic analysis. Prior studies have predominantly described the background color as pinkish-brown, violaceous, or dull pink to dull red [25]. In our cohort, the most frequently observed background was erythematous, followed by a violaceous hue. Linear vascular structures were the most frequently observed pattern, with occasional sea anemone-like arrangements. Other vascular configurations, such as radial, hairpin, and dotted vessels, were described but less frequently reported [31]. In the control group, buccal mucosal lesions were significantly different, with a predominance of globular and dotted patterns, while reticular striae (present in 90.9%, *n* = 30/33 of OLP cases) were almost absent. Linear striae were paradoxically more frequent in the controls than in OLP, highlighting the need to interpret striae morphology alongside other features. Moreover, diffuse regular vascular distribution and dotted vessels were significantly more common in controls, while radial vascular patterns were specific to OLP. Veil-like structures and leukoplakia-like lesions were also more frequently identified in controls, suggesting their lower specificity for OLP.

On the lingual mucosa, OLP typically manifests as white patches, erosions, or ulcers, particularly on the dorsal or lateral surfaces. The literature describes WS in circular, structureless, veil-like patterns, often appearing gray-white to bluish-white, commonly on the dorsum of the tongue. These lesions can include leukoplakia-like areas and a smoothing or blunting of lingual papillae. Erosions on the tongue are well-defined, bright red, and often surrounded by a hyperkeratotic white rim. Pigmentation may appear as brown or blue-gray clods and globules [32,33,34]. In our cohort, the most common background color was pink, followed by erythematous. The most prevalent white lesion was leukoplakia-like, suggesting a higher degree of hyperkeratosis in lingual OLP. Reticular striae were also present but at a lower frequency than in the buccal mucosa. A radial peripheral vascular pattern and dotted vessels were prominent, reinforcing the vascular remodeling characteristic of lingual OLP. Erosions were frequently observed, along with blunting of the lingual papillae—a finding absent in the control group. Compared with the control group for the lingual mucosa, distinct differences were evident. Globular patterns were overwhelmingly more common in hypertrophic candidiasis than OLP (*p* < 0.001), while linear and dotted vessels, radial vascular arrangements, and blunting of papillae were unique to OLP. This contrast supports the diagnostic potential of mucoscopy in distinguishing lingual OLP from candidiasis, which typically lacks structured white striae and presents with diffuse erythema or pseudomembranous plaques.

Mucoscopy has emerged as an essential non-invasive tool in the early detection and differentiation of squamous cell carcinoma (SCC) of the lip and oral mucosa. Given the challenges of distinguishing potentially malignant disorders and malignant lesions from benign inflammatory conditions, mucoscopy provides enhanced visualization of vascular and structural alterations that may indicate malignant transformation. One of the most significant mucoscopic findings in oral SCC is the presence of irregular, polymorphous, or atypical vascular patterns, reflecting tumor-induced neovascularization. Linear-irregular vessels, dotted vessels, and glomerular vessels are commonly observed in early SCC, whereas polymorphous vascular arrangements suggest progression toward malignancy. Additionally, white structureless areas and keratinization indicate hyperproliferative activity, which, when combined with ulcerations and a milky red or erythematous background, strongly raises suspicion for invasive SCC. In cases of OLP with suspected malignant transformation, the loss of Wickham’s striae, the appearance of disorganized pigmentation, and an increase in vascular density should prompt further histopathological evaluation. By integrating mucoscopy into routine oral examinations, clinicians can facilitate earlier biopsy decisions, improving early SCC detection and patient outcomes. Future studies should aim to standardize mucoscopic criteria for oral SCC and further investigate its role in monitoring high-risk lesions such as EOLP [35,36,37,38,39,40,41].

Our findings support the diagnostic utility of mucoscopy in the evaluation of OLP, especially in distinguishing it from clinically similar inflammatory oral conditions. This aligns with the conclusions of the American Academy of Oral and Maxillofacial Pathology, which emphasize the ongoing challenges in achieving a reliable diagnosis of OLP due to its overlapping clinical and histopathologic features with other lichenoid disorders and immune-mediated mucosal diseases. The current expert consensus emphasizes the necessity of thorough clinicopathologic correlation and ongoing follow-up, acknowledging the risk of disease progression or evolution into other lichenoid disorders. Within this framework, mucoscopy emerges as a valuable non-invasive diagnostic adjunct, capable of highlighting striae, vascular patterns, and erosions that may remain undetected during routine clinical examination. By identifying features such as leukoplakia-like plaques and associated vascular patterns, mucoscopy may contribute to earlier recognition of lesions with malignant potential and guide biopsy decisions. These observations support the call for more refined diagnostic approaches, as outlined by Cheng et al., and point to mucoscopy as a complementary tool to enhance both clinical accuracy and research reproducibility in OLP [42].

It is crucial to emphasize that, while mucoscopy exhibits high sensitivity and specificity in diagnosing OLP, it cannot currently serve as the sole method for a definitive diagnosis. This limitation stems from insufficient large-scale patient cohorts to validate mucoscopic criteria for conclusive diagnosis, as well as a lack of comparative studies between mucoscopic findings of OLP and similar conditions. Therefore, histopathological examination remains the gold standard for definitive diagnosis. Mucoscopy continues to serve as a primary non-invasive tool that supports clinical diagnosis and aids in monitoring the effectiveness of OLP treatments. It offers supplementary insights that assist clinicians in determining the necessity and the best site for biopsy or further evaluation. While reticular striae were highly specific to OLP in our study, particularly on the buccal mucosa (90.9%, *p* < 0.001), other mucoscopic features were rather important in association rather than individually. Thus, leukoplakia-like lesions were most prevalent on the lingual mucosa and significantly associated with dotted (*p* = 0.027) and looped vessels (*p* = 0.002). Erosions correlated significantly with both dotted (*p* < 0.001) and linear vessels (*p* = 0.011), especially in lingual and vermilion lesions.

This study has several limitations that should be acknowledged. The small sample size and single-center design may limit the generalizability of the findings, necessitating larger, multi-center studies for validation. Additionally, the study lacks longitudinal follow-up, preventing an assessment of mucoscopic changes over time and their potential role in predicting disease progression or malignant transformation. Another limitation is the potential for observer bias, as mucoscopic evaluations were performed by a limited number of examiners without interobserver agreement analysis. Future studies should focus on larger, prospective cohorts, direct histopathologic comparisons, and multi-observer evaluations to further refine the role of mucoscopy in OLP diagnosis and monitoring.

## 5. Conclusions

This study highlights the diagnostic value of mucoscopy in OLP, revealing site-specific patterns that contribute to distinguishing it from other oral inflammatory conditions. Buccal lesions predominantly showed classical reticular striae with minimal vascular changes, whereas vermilion and lingual sites exhibited more prominent vascular structures, scaling, and leukoplakia-like areas, suggesting increased inflammatory activity, chronicity, and epithelial alteration. Dotted and linear vessels were more frequently observed in erosive and hyperkeratotic lesions, supporting a potential role for angiogenesis in active disease. Compared to the control group, OLP lesions presented more structured and disease-specific features, while mimicking conditions showed diffuse, nonspecific patterns.

This study reinforces the diagnostic and prognostic role of mucoscopy in OLP, demonstrating site-specific mucoscopic variations that may aid in differentiating disease subtypes and identifying high-risk lesions. Future research should focus on longitudinal studies, standardized mucoscopic criteria, and AI-assisted image analysis to enhance the diagnostic accuracy and predictive value of mucoscopy in OLP and its malignant transformation.

## Figures and Tables

**Table 1:** Distribution of cases in the control group by diagnosis and mucosal site.

Mucosal Site	Diagnosis	No.
Vermilion (*n* = 30)	Chronic cheilitis Actinic Exfoliative Eczematous	
		20
		5
		3
	Lip squamous cell carcinoma	2
Buccal mucosa (*n* = 20)	Oral PV	15
	Hyperplastic oral candidiasis	3
	Morsicatio buccarum	2
Lingual mucosa (*n* = 30)	Hyperplastic oral candidiasis	25
	Oral leukoplakia	2
	Oral PV	2
	Pachyonychia congenita	1

**Table 2:** Demographic data of the study population.

		*n*/33	%
**Gender**	M	9	27.3
	F	24	72.7
**Age**	<30 ys	3	9.1
	31–50 ys	11	33.3
	51–70 ys	14	42.4
	>70 ys	5	15.2
**Total**		33	100.0

**Table 3:** Clinical features of the OLP study population.

		*n*/33	%
**Clinical aspect**	Erosive	15/33	45.5
	Reticular	18/33	54.5
**Associated cutaneous lesions**	No	19/33	57.6
	Yes	14/33	42.4
**Total**		33/33	100.0

**Table 4:** Overall distribution of the mucoscopic features in the OLP study cohort (*n* = 33).

Parameter Investigated		Number of Patients (*n*/33)	Percentages (%)
**Background color**	Erythematous	17/33	51.5
	Violaceous	11/33	33.3
**White lesions**	Radial striae	15/33	45.4
	Linear striae	28/33	84.8
	Reticular striae	31/33	93.9
	Leaf venation	7/33	21.2
	Annular striae	5/33	15.1
	Globular	6/33	18.1
	Dotted	5/33	15.1
	Veil	19/33	57.5
	Rosette	1/33	3.0
	Leukoplakia-like	20/33	60.6
**Blood vessels**	Linear	19/33	57.5
	Dotted	23/33	69.6
	Looped	8/33	24.2
	Sea anemone-like	1/33	3.0
**Distribution of blood vessels**	Radial at the perifery	21/33	63.6
	Diffuse regular	9/33	27.2
**Erosions**		16/33	48.4
**Scales**		14/33	42.4
**Blunting of lingual papillae**		20/33	60.6

**Table 5:** Mucoscopic features of OLP and distribution in different oral sites.

		Vermilion		Lingual		Buccal	
		*n*/33	%	*n*/33	%	*n*/33	%
**Background color**	Pink	23/33	69.7	21/33	63.6	30/33	90.9
	Erythematous	10/33	30.3	11/33	33.3	8/33	24.2
	Violaceous	9/33	27.3	3/33	9.1	-	-
**White lesions**	Radial striae	5/33	15.2	3/33	9.1	7/33	21.2
	Linear striae	27/33	81.8	4/33	12.1	7/33	21.2
	Reticular striae	13/33	39.4	6/33	18.2	30/33	90.9
	Leaf venation	1/33	3.0	-	-	6/33	18.2
	Annular striae	3/33	9.1	2/33	6.1	1/33	3.0
	Globular	2/33	6.1	4/33	12.1	1/33	3.0
	Dotted	1/33	3.0	1/33	3.0	3/33	9.1
	Veil	5/33	15.2	14/33	42.4	7/33	21.2
	Rosette	1/33	3.0	-	-	-	-
	Leukoplakia-like	6/33	18.2	16/33	48.5	5/33	15.2
**Blood vessels**	Linear	13/33	39.4	12/33	36.4	5/33	15.2
	Dotted	19/33	57.6	12/33	36.4	5/33	15.2
	Looped	5/33	15.2	4/33	12.1	2/33	6.1
	Sea anemone-like	-	-	1/33	3.0	1/33	3.0
**Distribution of blood vessels**	Radial at the perifery	16/33	48.5	16/33	48.5	7/33	21.2
	Diffuse regular	10/33	30.3	2/33	6.1	2/33	6.1
**Erosions**		13/33	39.4	14/33	42.4	12/33	36.4
**Scales**		16/33	48.5	-	-	-	-
**Blunting of lingual papillae**		0	0	20/33	60.6	-	-

**Table 6:** Mucoscopic features of OLP compared to the control group on the vermilion border of the lips.

	Group				Total		Pearson Chi-Squared Test *p*-Value
	OLP		Control				
	N	%	N	%	N	%	
**Background color**							
Erythematous	10	30.3%	24	80.0%	34	54.0%	<0.001 **
Violaceous	9	27.3%	9	30.0%	18	28.6%	0.811
**White lesions**							
Radial striae	5	15.2%	4	13.3%	9	14.3%	1.000
Linear striae	27	81.8%	20	66.7%	47	74.6%	0.168
Reticular striae	13	39.4%	-	-	13	20.6%	<0.001 **
Leaf venation	1	3.0%	-	-	1	1.6%	1.000
Annular striae	3	9.1%	-	-	3	4.8%	0.240
Globular	2	6.1%	9	30.0%	11	17.5%	0.012 *
Dotted	1	3.0%	6	20.0%	7	11.1%	0.052
Veil	5	15.2%	20	66.7%	25	39.7%	<0.001 **
Rosette	1	3.0%	-	-	1	1.6%	1.000
Leukoplakia-like	6	18.2%	15	50.0%	21	33.3%	0.007 **
**Blood vessels**							
Linear	13	39.4%	12	40.0%	25	39.7%	0.961
Dotted	19	57.6%	23	76.7%	42	66.7%	0.108
Looped	5	15.2%	4	13.3%	9	14.3%	1.000
Sea anemone-like	-	-	1	3.3%	1	1.6%	0.476
**Distribution of blood vessels**							
Radial at the perifery	16	48.5%	9	30.0%	25	39.7%	0.134
Diffuse regular	10	30.3%	18	60.0%	28	44.4%	0.018 *
**Erosions**	13	39.4%	16	53.3%	29	46.0%	0.268
**Scales**	16	48.5%	30	100.0%	46	73.0%	<0.001 **

* Statistically significant, ** statistically highly significant.

**Table 7:** Mucoscopic features of OLP compared to the control group on the lingual mucosa.

	Group				Total		Pearson Chi-Squared Test *p*-Value
	OLP		Control				
	N	%	N	%	N	%	
**Background color**							
Erythematous	11	33.3%	21	70.0%	32	50.8%	0.004 **
Violaceous	3	9.1%	-	-	3	4.8%	0.240
**White lesions**							
Radial striae	3	9.1%	1	3.3%	4	6.3%	0.614
Linear striae	4	12.1%	2	6.7%	6	9.5%	0.674
Reticular striae	6	18.2%	1	3.3%	7	11.1%	0.107
Leaf venation	-	-	-	-	-	-	
Annular striae	2	6.1%	-	-	2	3.2%	0.493
Globular	4	12.1%	28	93.3%	32	50.8%	<0.001 **
Dotted	1	3.0%	1	3.3%	2	3.2%	1.000
Veil	14	42.4%	6	20.0%	20	31.7%	0.056
Rosette	-	-	-	-	-	-	
Leukoplakia-like	16	48.5%	9	30.0%	25	39.7%	0.134
**Blood vessels**							
Linear	12	36.4%	-	-	12	19.0%	<0.001 **
Dotted	12	36.4%	1	3.3%	13	20.6%	0.001 **
Looped	4	12.1%	-	-	4	6.3%	0.115
Sea anemone-like	1	3.0%	-	-	1	1.6%	1.000
**Distribution of blood vessels**							
Radial at the perifery	16	48.5%	-	-	16	25.4%	<0.001 **
Diffuse regular	2	6.1%	1	3.3%	3	4.8%	1.000
**Erosions**	14	42.4%	5	16.7%	19	30.2%	0.026 *
**Blunting of lingual papillae**	20	60.6%	3	10.0%	23	36.5%	<0.001 **

* Statistically significant, ** statistically highly significant.

**Table 8:** Mucoscopic features of OLP compared to the control group on the buccal mucosa.

	Group				Total		Pearson Chi-Squared Test *p*-Value
	OLP		Control				
	N	%	N	%	N	%	
**Background color**							
Erythematous	8	24.2%	17	85.0%	25	47.2%	<0.001 **
Violaceous	-	-	-	-	-	-	
**White lesions**							
Radial striae	7	21.2%	5	25.0%	12	22.6%	0.748
Linear striae	7	21.2%	18	90.0%	25	47.2%	<0.001 **
Reticular striae	30	90.9%	1	5.0%	31	58.5%	<0.001 **
Leaf venation	6	18.2%	-	-	6	11.3%	0.072
Annular striae	1	3.0%	-	-	1	1.9%	1.000
Globular	1	3.0%	15	75.0%	16	30.2%	<0.001 **
Dotted	3	9.1%	9	45.0%	12	22.6%	0.005 **
Veil	7	21.2%	11	55.0%	18	34.0%	0.012 **
Rosette	-	-	-	-	-	-	
Leukoplakia-like	5	15.2%	6	30.0%	11	20.8%	0.296
**Blood vessels**							
Linear	5	15.2%	1	5.0%	6	11.3%	0.390
Dotted	5	15.2%	13	65.0%	18	34.0%	<0.001 **
Looped	2	6.1%	1	5.0%	3	5.7%	1.000
Sea anemone-like	1	3.0%	-	-	1	1.9%	1.000
**Distribution of blood vessels**							
Radial at the perifery	7	21.2%	-	-	7	13.2%	0.037 *
Diffuse regular	2	6.1%	13	65.0%	15	28.3%	<0.001 **
**Erosions**	12	36.4%	15	75.0%	27	50.9%	0.006 **

* Statistically significant, ** statistically highly significant.

## Data Availability

The original contributions presented in this study are included in the article. Further inquiries can be directed to the corresponding authors.

## Abbreviations

The following abbreviations are used in this manuscript:

OLP	Oral Lichen Planus
LP	Lichen Planus
HCV	Hepatitis C Virus
EOLP	Erosive Oral Lichen Planus
WHO	World Health Organization
DIF	Direct Immunofluorescence
SD	Standard Deviation
PV	Pemphigus Vulgaris
AI	Artificial Intelligence
SCC	Squamous Cell Carcinoma

## Appendix A

**Table A1:** The association between white lesions and blood vessel patterns of the vermilion.

VERMILION		BLOOD VESSELS															
WHITE LESIONS		Linear				Dotted				Looped				Sea Anemone-Like			
		0		1		0		1		0		1		0		1	
		N	%	N	%	N	%	N	%	N	%	N	%	N	%	N	%
Radial striae	0	17	85.0%	11	84.6%	14	100.0%	14	73.7%	24	85.7%	4	80.0%	28	84.8%	-	-
	1	3	15.0%	2	15.4%			5	26.3%	4	14.3%	1	20.0%	5	15.2%	-	-
		*p* = 1.000				*p* = 0.057 *				*p* = 1.000				-			
Linear striae	0	5	25.0%	1	7.7%	5	35.7%	1	5.3%	6	21.4%			6	18.2%	-	-
	1	15	75.0%	12	92.3%	9	64.3%	18	94.7%	22	78.6%	5	100.0%	27	81.8%	-	-
		*p* = 0.364				*p* = 0.062				*p* = 0.556				-			
Reticular striae	0	13	65.0%	7	53.8%	6	42.9%	14	73.7%	16	57.1%	4	80.0%	20	60.6%	-	-
	1	7	35.0%	6	46.2%	8	57.1%	5	26.3%	12	42.9%	1	20.0%	13	39.4%	-	-
		*p* = 0.522				*p* = 0.073				*p* = 0.625				-			
Leaf venation	0	20	100.0%	12	92.3%	14	100.0%	18	94.7%	28	100.0%	4	80.0%	32	97.0%	-	-
	1			1	7.7%			1	5.3%			1	20.0%	1	3.0%	-	-
		*p* = 0.394				*p* = 1.000				*p* = 0.152				-			
Annular striae	0	18	90.0%	12	92.3%	14	100.0%	16	84.2%	27	96.4%	3	60.0%	30	90.9%	-	-
	1	2	10.0%	1	7.7%			3	15.8%	1	3.6%	2	40.0%	3	9.1%	-	-
		*p* = 1.000				*p* = 0.244				*p* = 0.053 *				-			
Globular	0	19	95.0%	12	92.3%	14	100.0%	17	89.5%	27	96.4%	4	80.0%	31	93.9%	-	-
	1	1	5.0%	1	7.7%			2	10.5%	1	3.6%	1	20.0%	2	6.1%	-	-
		*p* = 1.000				*p* = 0.496				*p* = 0.284				-			
Dotted	0	20	100.0%	12	92.3%	14	100.0%	18	94.7%	27	96.4%	5	100.0%	32	97.0%	-	-
	1			1	7.7%			1	5.3%	1	3.6%			1	3.0%	-	-
		*p* = 0.394				*p* = 1.000				*p* = 1.000				-			
Veil	0	16	80.0%	12	92.3%	11	78.6%	17	89.5%	24	85.7%	4	80.0%	28	84.8%	-	-
	1	4	20.0%	1	7.7%	3	21.4%	2	10.5%	4	14.3%	1	20.0%	5	15.2%	-	-
		*p* = 0.625				*p* = 0.628				*p* = 1.000				-			
Rosette	0	19	95.0%	13	100.0%	14	100.0%	18	94.7%	27	96.4%	5	100.0%	32	97.0%	-	-
	1	1	5.0%					1	5.3%	1	3.6%			1	3.0%	-	-
		*p* = 1.000				*p* = 1.000				*p* = 1.000				-			
Leukoplakia-like	0	16	80.0%	11	84.6%	14	100.0%	13	68.4%	25	89.3%	2	40.0%	27	81.8%	-	-
	1	4	20.0%	2	15.4%			6	31.6%	3	10.7%	3	60.0%	6	18.2%	-	-
		*p* = 1.000				*p* = 0.027 *				*p* = 0.031 *				-			
**Total**		**20**	**100.0%**	**13**	**100.0%**	**14**	**100.0%**	**19**	**100.0%**	**28**	**100.0%**	**5**	**100.0%**	**33**	**100.0%**	**-**	**-**

Pearson Chi-squared test. * Statistically significant.

**Table A2:** Association between vermilion erosions and blood vessels.

VERMILION		BLOOD VESSELS															
EROSION		Linear				Dotted				Looped				Sea Anemone-Like			
		0		1		0		1		0		1		0		1	
		N	%	N	%	N	%	N	%	N	%	N	%	N	%	N	%
	0	17	85.0%	3	23.1%	12	85.7%	8	42.1%	19	67.9%	1	20.0%	20	60.6%	-	-
	1	3	15.0%	10	76.9%	2	14.3%	11	57.9%	9	32.1%	4	80.0%	13	39.4%	-	-
		*p* <0.001 **				*p* = 0.011 **				*p* = 0.066 *				-			
**Total**		**20**	**100.0%**	**13**	**100.0%**	**14**	**100.0%**	**19**	**100.0%**	**28**	**100.0%**	**5**	**100.0%**	**33**	**100.0%**	**-**	**-**

Pearson Chi-squared test. * Statistically significant, ** statistically highly significant.

**Table A3:** Association between white lesions and blood vessel patterns of the lingual mucosa.

LINGUAL		BLOOD VESSELS															
WHITE LESIONS		Linear				Dotted				Looped				Sea Anemone-Like			
		0		1		0		1		0		1		0		1	
		N	%	N	%	N	%	N	%	N	%	N	%	N	%	N	%
Radial striae	0	20	95.2%	10	83.3%	20	95.2%	10	83.3%	27	93.1%	3	75.0%	29	90.6%	1	100.0%
	1	1	4.8%	2	16.7%	1	4.8%	2	16.7%	2	6.9%	1	25.0%	3	9.4%		
		*p* = 0.538				*p* = 0.538				*p* = 0.330				*p* = 1.000			
Linear striae	0	20	95.2%	9	75.0%	19	90.5%	10	83.3%	26	89.7%	3	75.0%	28	87.5%	1	100.0%
	1	1	4.8%	3	25.0%	2	9.5%	2	16.7%	3	10.3%	1	25.0%	4	12.5%		
		*p* = 0.125				*p* = 0.610				*p* = 0.420				*p* = 1.000			
Reticular striae	0	19	90.5%	8	66.7%	19	90.5%	8	66.7%	24	82.8%	3	75.0%	26	81.3%	1	100.0%
	1	2	9.5%	4	33.3%	2	9.5%	4	33.3%	5	17.2%	1	25.0%	6	18.8%		
		*p* = 0.159				*p* = 0.159				*p* = 1.000				*p* = 1.000			
Leaf venation	0	21	100.0%	12	100.0%	21	100.0%	12	100.0%	29	100.0%	4	100.0%	32	100.0%	1	100.0%
	1	-	-	-	-	-	-	-	-	-	-	-	-	-	-	-	-
		-				-				-				-			
Annular striae	0	21	100.0%	10	83.3%	20	95.2%	11	91.7%	28	96.6%	3	75.0%	30	93.8%	1	100.0%
	1			2	16.7%	1	4.8%	1	8.3%	1	3.4%	1	25.0%	2	6.3%		
		*p* = 0.125				*p* = 1.000				*p* = 0.231				*p* = 1.000			
Globular	0	19	90.5%	10	83.3%	18	85.7%	11	91.7%	25	86.2%	4	100.0%	28	87.5%	1	100.0%
	1	2	9.5%	2	16.7%	3	14.3%	1	8.3%	4	13.8%			4	12.5%		
		*p* = 0.610				*p* = 1.000				*p* = 1.000				*p* = 1.000			
Dotted	0	20	95.2%	12	100.0%	21	100.0%	11	91.7%	29	100.0%	3	75.0%	31	96.9%	1	100.0%
	1	1	4.8%					1	8.3%			1	25.0%	1	3.1%		
		*p* = 1.000				*p* = 0.364				*p* = 0.121				*p* = 1.000			
Veil	0	14	66.7%	5	41.7%	14	66.7%	5	41.7%	19	65.5%			19	59.4%		
	1	7	33.3%	7	58.3%	7	33.3%	7	58.3%	10	34.5%	4	100.0%	13	40.6%	1	100.0%
		*p* = 0.162				*p* = 0.162				*p* = 0.024 *				*p* = 0.424			
Rosette	0	21	100.0%	12	100.0%	21	100.0%	12	100.0%	29	100.0%	4	100.0%	32	100.0%	1	100.0%
	1	-	-	-	-	-	-	-	-	-	-	-	-	-	-	-	-
		-				-				-				-			
Leukoplakia-like	0	14	66.7%	3	25.0%	15	71.4%	2	16.7%	17	58.6%			17	53.1%		
	1	7	33.3%	9	75.0%	6	28.6%	10	83.3%	12	41.4%	4	100.0%	15	46.9%	1	100.0%
		*p* = 0.021 *				*p* = 0.002 **				*p* = 0.044 *				*p* = 0.485			
**Total**		**21**	**100.0%**	**12**	**100.0%**	**21**	**100.0%**	**12**	**100.0%**	**29**	**100.0%**	**4**	**100.0%**	**32**	**100.0%**	**1**	**100.0%**

Pearson Chi-squared test. * Statistically significant, ** statistically highly significant.

**Table A4:** Association between lingual erosions and blood vessels.

LINGUAL		BLOOD VESSELS															
EROSION		Linear				Dotted				Looped				Sea Anemone-Like			
		0		1		0		1		0		1		0		1	
		N	%	N	%	N	%	N	%	N	%	N	%	N	%	N	%
	0	17	81.0%	2	16.7%	17	81.0%	2	16.7%	18	62.1%	1	25.0%	19	59.4%		
	1	4	19.0%	10	83.3%	4	19.0%	10	83.3%	11	37.9%	3	75.0%	13	40.6%	1	100.0%
		*p* < 0.001 **				*p* < 0.001 **				*p* = 0.288				*p* = 0.424			
**Total**		**21**	**100.0%**	**12**	**100.0%**	**21**	**100.0%**	**12**	**100.0%**	**29**	**100.0%**	**4**	**100.0%**	**32**	**100.0%**	**1**	**100.0%**

Pearson Chi-squared test. ** statistically highly significant.

**Table A5:** Association between white lesions and blood vessel patterns of the buccal mucosa.

BUCCAL		BLOOD VESSELS															
WHITE LESIONS		Linear				Dotted				Looped				Sea Anemone-Like			
		0		1		0		1		0		1		0		1	
		N	%	N	%	N	%	N	%	N	%	N	%	N	%	N	%
Radial striae	0	23	82.1%	3	60.0%	22	78.6%	4	80.0%	24	77.4%	2	100.0%	26	81.3%		
	1	5	17.9%	2	40.0%	6	21.4%	1	20.0%	7	22.6%			6	18.8%	1	100.0%
		*p* = 0.282				*p* = 1.000				*p* = 1.000				*p* = 0.212			
Linear striae	0	23	82.1%	3	60.0%	23	82.1%	3	60.0%	26	83.9%			26	81.3%		
	1	5	17.9%	2	40.0%	5	17.9%	2	40.0%	5	16.1%	2	100.0%	6	18.8%	1	100.0%
		*p* = 0.282				*p* = 0.282				*p* = 0.040 *				*p* = 0.212			
Reticular striae	0	1	3.6%	2	40.0%	3	10.7%			2	6.5%	1	50.0%	3	9.4%		
	1	27	96.4%	3	60.0%	25	89.3%	5	100.0%	29	93.5%	1	50.0%	29	90.6%	1	100.0%
		*p* = 0.053 *				*p* = 1.000				*p* = 0.176				*p* = 1.000			
Leaf venation	0	22	78.6%	5	100.0%	22	78.6%	5	100.0%	25	80.6%	2	100.0%	26	81.3%	1	100.0%
	1	6	21.4%			6	21.4%			6	19.4%			6	18.8%		
		*p* = 0.556				*p* = 0.556				*p* = 1.000				*p* = 1.000			
Annular striae	0	28	100.0%	4	80.0%	27	96.4%	5	100.0%	31	100.0%	1	50.0%	31	96.9%	1	100.0%
	1			1	20.0%	1	3.6%					1	50.0%	1	3.1%		
		*p* = 0.152				*p* = 1.000				*p* = 0.061 *				*p* = 1.000			
Globular	0	28	100.0%	4	80.0%	27	96.4%	5	100.0%	31	100.0%	1	50.0%	31	96.9%	1	100.0%
	1			1	20.0%	1	3.6%					1	50.0%	1	3.1%		
		*p* = 0.152				*p* = 1.000				*p* = 0.061 *				*p* = 1.000			
Dotted	0	26	92.9%	4	80.0%	25	89.3%	5	100.0%	29	93.5%	1	50.0%	29	90.6%	1	100.0%
	1	2	7.1%	1	20.0%	3	10.7%			2	6.5%	1	50.0%	3	9.4%		
		*p* = 0.400				*p* = 1.000				*p* = 0.176				*p* = 1.000			
Veil	0	22	78.6%	4	80.0%	24	85.7%	2	40.0%	25	80.6%	1	50.0%	26	81.3%		
	1	6	21.4%	1	20.0%	4	14.3%	3	60.0%	6	19.4%	1	50.0%	6	18.8%	1	100.0%
		*p* = 1.000				*p* = 0.052 *				*p* = 0.384				*p* = 0.212			
Rosette	0	28	100.0%	5	100.0%	28	100.0%	5	100.0%	31	100.0%	2	100.0%	32	100.0%	1	100.0%
	1	-	-	-	-	-	-	-	-	-	-	-	-	-	-	-	-
		-				-				-				-			
Leukoplakia-like	0	25	89.3%	3	60.0%	25	89.3%	3	60.0%	27	87.1%	1	50.0%	27	84.4%	1	100.0%
	1	3	10.7%	2	40.0%	3	10.7%	2	40.0%	4	12.9%	1	50.0%	5	15.6%		
		*p* = 0.155				*p* = 0.155				*p* = 0.284				*p* = 1.000			
**Total**		**28**	**100.0%**	**5**	**100.0%**	**28**	**100.0%**	**5**	**100.0%**	**31**	**100.0%**	**2**	**100.0%**	**32**	**100.0%**	**1**	**100.0%**

Pearson Chi-squared test. * Statistically significant.

**Table A6:** Association between buccal erosions and blood vessels.

BUCCAL		BLOOD VESSELS															
EROSION		Linear				Dotted				Looped				Sea Anemone-Like			
		0		1		0		1		0		1		0		1	
		N	%	N	%	N	%	N	%	N	%	N	%	N	%	N	%
	0	21	75.0%			19	67.9%	2	40.0%	20	64.5%	1	50.0%	21	65.6%		
	1	7	25.0%	5	100.0%	9	32.1%	3	60.0%	11	35.5%	1	50.0%	11	34.4%	1	100.0%
		*p* = 0.003 **				*p* = 0.328				*p* = 1.000				*p* = 0.364			
**Total**		**28**	**100.0%**	**5**	**100.0%**	**28**	**100.0%**	**5**	**100.0%**	**31**	**100.0%**	**2**	**100.0%**	**32**	**100.0%**	**1**	**100.0%**

Pearson Chi-squared test. ** statistically highly significant.

## References

[1] Warnakulasuriya S. (2018). Clinical Features and Presentation of Oral Potentially Malignant Disorders. Oral Surg. Oral Med. Oral Pathol. Oral Radiol..

[2] Lavanya N., Jayanthi P., Rao U.K., Ranganathan K. (2011). Oral lichen planus: An update on pathogenesis and treatment. J. Oral Maxillofac. Pathol..

[3] Li C., Tang X., Zheng X., Ge S., Wen H., Lin X., Chen Z., Lu L. (2020). Global Prevalence and Incidence Estimates of Oral Lichen Planus: A Systematic Review and Meta-analysis. JAMA Dermatol..

[4] Mohan R.P.S., Gupta A., Kamarthi N., Malik S., Goel S., Gupta S. (2017). Incidence of Oral Lichen Planus in Perimenopausal Women: A Cross-sectional Study in Western Uttar Pradesh Population. J. Midlife Health.

[5] Arduino P.G., Magliano A., Gambino A., Macciotta A., Carbone M., Conrotto D., Karimi D., Carrozzo M., Broccoletti R. (2021). Risk of Malignant Transformation in 3173 Subjects with Histopathologically Confirmed Oral Lichen Planus: A 33-Year Cohort Study in Northern Italy. Cancers.

[6] González-Moles M.Á., Ramos-García P. (2024). An Evidence-Based Update on the Potential for Malignancy of Oral Lichen Planus and Related Conditions: A Systematic Review and Meta-Analysis. Cancers.

[7] Manchanda Y., Rathi S.K., Joshi A., Das S. (2023). Oral Lichen Planus: An Updated Review of Etiopathogenesis, Clinical Presentation, and Management. Indian Dermatol. Online J..

[8] Adnane S., Mahad C., Haitami S., Yahya I.B. (2022). Hepatitis C virus infection and oral lichen planus: A controversial association. Adv. Oral Maxillofac. Surg..

[9] Rahat S., Kashetsky N., Bagit A., Sachdeva M., Lytvyn Y., Mufti A., Maibach H.I., Yeung J. (2021). Can We Separate Oral Lichen Planus from Allergic Contact Dermatitis and Should We Patch Test? A Systematic Review of Chronic Oral Lichenoid Lesions. Dermatitis.

[10] Teoh L., Moses G., McCullough M.J. (2019). A review and guide to drug-associated oral adverse effects—Oral mucosal and lichenoid reactions. Part 2. J. Oral Pathol. Med..

[11] Gholizadeh N., Sadeghi A., Mirzaii-Dizgah I., Sheykhbahaei N. (2021). Serum level of estrogen in Iranian patients with oral lichen planus. Asian Biomed. (Res. Rev. News).

[12] Ślebioda Z., Drożdżyńska J., Karpińska A., Krzyżaniak A., Kasperczak M., Tomoń N., Wiśniewska P., Wyganowska M.L. (2024). Oral Lichen Planus: Clinical Presentation, Demographic Characteristics, and Risk Factors in a Retrospective Study of 186 Polish Patients. J. Clin. Med..

[13] Arnold D.L., Krishnamurthy K. (2025). Lichen Planus. StatPearls.

[14] GunaShekhar M., Sudhakar R., Shahul M., Tenny J., Ravikanth M., Manikyakumar N. (2010). Oral lichen planus in childhood: A rare case report. Dermatol. Online J..

[15] Gall R., Navarro-Fernandez I.N. (2025). Lichen Planus Erosive Form. StatPearls.

[16] Tampa M., Mitran M., Mitran C., Sarbu I., Rusu L.-C., Matei C., Constantin C., Neagu M., Georgescu S.-R. (2018). Markers of Oral Lichen Planus Malignant Transformation. Dis. Markers.

[17] Idrees M., Kujan O., Shearston K., Farah C.S. (2021). Oral lichen planus has a very low malignant transformation rate: A systematic review and meta-analysis using strict diagnostic and inclusion criteria. J. Oral Pathol. Med..

[18] Van Der M.E.H., Van Der W.I. (2003). Lack of clinicopathologic correlation in the diagnosis of oral lichen planus based on the presently available diagnostic criteria and suggestions for modifications. J. Oral Pathol. Med..

[19] Batra M.R., Mohod S., Sawarbandhe P. (2024). Oral Lichen Planus and Its Therapeutic Approaches: A Case Report. Cureus.

[20] Litaiem N., Mansour Y., Jones M., Zeglaoui F. (2016). Dermoscopic signs of lichen planus. BMJ Case Rep..

[21] Buajeeb W., Okuma N., Thanakun S., Laothumthut T. (2015). Direct Immunofluorescence in Oral Lichen Planus. J. Clin. Diagn. Res..

[22] Yu S., Sun W., Mi D., Jin S., Wu X., Xin B., Zhang H., Wang Y., Sun X., He X. (2024). Artificial Intelligence Diagnosing of Oral Lichen Planus: A Comparative Study. Bioengineering.

[23] Achararit P., Manaspon C., Jongwannasiri C., Phattarataratip E., Osathanon T., Sappayatosok K. (2023). Artificial Intelligence-Based Diagnosis of Oral Lichen Planus Using Deep Convolutional Neural Networks. Eur. J. Dent..

[24] Chen X., Lu Q., Chen C., Jiang G. (2021). Recent developments in dermoscopy for dermatology. J. Cosmet. Dermatol..

[25] Ashok S. (2023). Dermatoscopy: A New Diagnostic Approach for Lesions on Mucous Membrane. Clinical Diagnosis and Management of Squamous Cell Carcinoma.

[26] Rouai M., Litaiem N., Hammami H., Bacha T., Jones M., Ksontini M., Rammeh S., Mokni M., Zeglaoui F. (2021). Dermoscopic features of mucosal lichen planus. Int. J. Dermatol..

[27] Singh S.K., Kharabanda A., Kundalia A. (2025). Dermoscopic features in different types of lichen planus: A case series. Int. J. Res. Dermatol..

[28] Humberto J.S.M., Saia R.S., Costa L.H.A., Rocha M.J.A., Motta A.C.F. (2024). Salivary cytokine profile in patients with oral lichen planus. Odovtos Int. J. Dent. Sci..

[29] Popa C., Sciuca A.M., Onofrei B.-A., Toader S., Hritcu O.M.C., Colac C.B., Andrese E.P., Brănișteanu D.E., Toader M.P. (2024). Integrative Approaches for the Diagnosis and Management of Erosive Oral Lichen Planus. Diagnostics.

[30] Mittal S., Vinitha N.M., Chaitra V. (2022). Dermoscopy of Isolated Lip Lichen Planus. Indian Dermatol. Online J..

[31] Marcu C.A., Parlatescu I., Tovaru S., Nicolae C.L., Costache M., Tovaru M. (2024). Lichen Planus of the Lip—Case Series and Review of the Literature. Medicina.

[32] Rather S., Shah A.A., Shah F.Y., S K., Bhat M.A., Reyaz S., Hassan I. (2022). Dermoscopy of Oral Mucosal Lesions: Experience from a Tertiary Care Center in North India and Review of Literature. Indian Dermatol. Online J..

[33] Sachdeva S., Sachdeva S., Kapoor P. (2011). Wickham striae: Etiopathogenensis and clinical significance. Indian J. Dermatol..

[34] Makhecha M., Singh T., Malladi N., Rambhia K. (2020). Dermoscopic features of various stages of lichen planus. Indian J. Dermatol. Venereol. Leprol..

[35] Sonthalia S., Varma S., Jha A.K., Jakhar D., Kaliyadan F. (2018). Case Report: Dermoscopic features of oral lichen planus—The evolution of mucoscopy. F1000Research.

[36] Jha A.K., Vinay K., Sławińska M., Sonthalia S., Sobjanek M., Kamińska-Winciorek G., Errichetti E., Kamat D., Chatterjee D., Apalla Z. (2021). Application of mucous membrane dermoscopy (mucoscopy) in diagnostics of benign oral lesions—Literature review and preliminary observations from International Dermoscopy Society study. Dermatol. Ther..

[37] Sousa F., Blumer Rosa L. (2008). Oral lichen planus: Clinical and histopathological considerations. Braz. J. Otorhinolaryngol..

[38] Jose S.C., Kurien G.V. (2020). Diagnostic dermoscopic features and the correlation between dermoscopic and histopathologic features in lichen planus. Int. J. Res. Dermatol..

[39] Peralta R., Salerni G., Sabban E.C., Marin M.B., Cabo H. (2019). Dermoscopy of a Squamous Cell Carcinoma of the Lower Lip Showing Multiple Rosettes. Dermatol. Pract. Concept..

[40] Lallas A., Martínez G., Arceu M., Kyrgidis A., Liopyris K., Brancaccio G., Longo C., Errichetti E., Sgouros D., Papageorgiou C. (2022). Clinical and dermatoscopic predictors of squamous cell carcinoma of the lips: A case-control, multicentric study. J. Eur. Acad. Dermatol. Venereol..

[41] Tsushima F., Sakurai J., Uesugi A., Oikawa Y., Ohsako T., Mochizuki Y., Hirai H., Kayamori K., Harada H. (2021). Malignant transformation of oral lichen planus: A retrospective study of 565 Japanese patients. BMC Oral Health.

[42] Cheng Y.S., Gould A., Kurago Z., Fantasia J., Muller S. (2016). Diagnosis of oral lichen planus: A position paper of the American Academy of Oral and Maxillofacial Pathology. Oral Surg. Oral Med. Oral Pathol. Oral Radiol..

